# Gender and Uveitis in Patients with Multiple Sclerosis

**DOI:** 10.1155/2014/565262

**Published:** 2014-05-07

**Authors:** Lynn K. Gordon, Debra A. Goldstein

**Affiliations:** ^1^Department of Ophthalmology, Jules Stein Eye Institute, David Geffen School of Medicine at UCLA, Los Angeles, CA 90095, USA; ^2^Department of Ophthalmology, Northwestern Feinberg School of Medicine, Chicago, IL 60613, USA

## Abstract

Multiple sclerosis (MS), a demyelinating disease of the central nervous system, is more commonly seen in women. It has been associated with both anterior and intermediate uveitis as well as retinal vasculitis. Ocular inflammation may develop concurrent with, prior to, or after the development of neurologic signs and symptoms. Patients with MS have an approximately 1% chance of developing intraocular inflammation. Patients with intermediate uveitis have an 8–12% risk of being diagnosed with MS. This risk is higher in females and in those with bilateral disease. This should be kept in mind when evaluating patients with uveitis, particularly in those patients for whom TNF inhibitor therapy is being considered, as these agents may worsen demyelinating disease.

## 1. Introduction

Multiple sclerosis (MS), a chronic and episodic demyelinating disease of the central nervous system, affects many aspects of vision and ocular health, from afferent to efferent pathways. Ocular signs and symptoms associated with MS range from optic neuritis, the most commonly associated ophthalmologic disease, which occurs in nearly 50% of MS patients, to internuclear ophthalmoplegia, associated with demyelinating lesions of the medial longitudinal fasciculus, and to various types of debilitating nystagmus and, less commonly, ocular inflammatory disease [1–4]. Disability secondary to visual symptoms is an important cause of morbidity among patients with MS [5]. A large sampling of self-reported data from almost 9000 North American patients with MS revealed that nearly 16% of patients reported a visual comorbidity, most occurring after the diagnosis of MS was made [5]. Uveitis was identified by 3% of these individuals as responsible for significant disease morbidity. In this paper we highlight gender differences in MS and review the data regarding MS-associated uveitis.

## 2. Gender Differences in Prevalence/Incidence

Autoimmune diseases are a significant cause of morbidity and mortality, which disproportionately affect young and middle-aged women [6]. For MS, the female to male ratio is estimated to be approximately 3.5 : 1, and the ratio of affected women to affected men has actually increased over the past several decades [7]. The prevalence is estimated to be around 1 per 100,000 individuals. Although the mean age for diagnosis of MS is around 37, the typical age of onset ranges from the early 20s to the mid 40s. However, MS can also occur in children and in older adults; it is just less commonly diagnosed at the extremes of life.

There are geographic differences in disease onset, typically with higher incidence in countries further away from the equator and a lower incidence in Asia, possibly implicating environmental factors or infectious agents in disease pathogenesis [8]. However, there are exceptions to this observation and this generalization is not completely valid. Some populations are at high risk in countries close to the equator and others are at low risk in certain northern countries [9]. The risk for disease in these countries may reflect other genetic, epigenetic, or noninfectious environmental factors.

In addition to differences in rates of development of MS between men and women, there are differences between the sexes in age at development of the disease and, possibly, in disease course. One large series reported that MS presented, on average, two years later in men than women (31.2 years versus 29.3 years) [10]. As well, there is evidence from some series that males are more likely to develop primary-progressive MS, although this has not been borne out by a large meta-analysis [11].

Genetic susceptibility factors also play an important role, with an increased concordance of disease among monozygotic twins as compared to dizygotic twins, as well as an increased risk for MS among first-degree relatives of patients with the disease. Risk loci in the human leukocyte antigen (HLA) family of molecules including HLA-DRB1 have been implicated in disease susceptibility [7, 12, 13]. Large genome wide association studies (GWAS) demonstrated additional complex genetic candidates for disease susceptibility; these candidates typically involve genes in immunologic pathways including T-cell differentiation [7].

## 3. Clinical Manifestations of Inflammatory Ocular Disease

The clinical manifestations of intraocular inflammation in MS include chronic anterior uveitis, intermediate uveitis, and retinal vasculitis [14–18]. In addition to vasculitis and ischemia, granulomatous retinal periphlebitis was found in 4 cases (7 eyes) and focal lymphocytic or granulomatous retinitis was present in 3 cases (5 eyes) of 47 autopsy cases of multiple sclerosis studied pathologically [19]. Complications of inflammation include cataract, elevation in intraocular pressure, hypotony, retinal ischemia, and retinal neovascularization. In one retrospective study, the authors described 16 patients seen in a uveitis service who carried a diagnosis of MS [14]. They observed anterior uveitis in about 30% of eyes and intermediate uveitis and posterior uveitis each in about 20% of eyes. Three of the patients were not known to have MS at the time of their ocular disease onset. In a different study, 3.1% of 1916 uveitis patients seen in a tertiary care clinic had a diagnosis of MS [20]. In this study anterior uveitis accounted for 10% and intermediate uveitis comprised 78% of the uveitis seen in patients with MS; posterior and panuveitis were each seen in a single patient. Of those with both MS and uveitis, 74.6% were female, highlighting the gender disparity of the underlying autoimmune disease.

Another study of 1254 patients with uveitis revealed 16 with a concomitant diagnosis of MS, for a prevalence of 1.3%; the majority of these patients had bilateral disease [21]. Nine of these patients had diagnoses of MS that preceded onset of uveitis, three patients had concurrent diagnoses, and four patients developed uveitis prior to the diagnosis of MS. 88% of these patients were female. The mean age for the diagnosis of uveitis was 37.2 and mean age for diagnosis of MS was 35.5 years. Almost half of these patients also had optic neuritis. Granulomatous anterior uveitis was present in 56%, cataract in 38%, intermediate uveitis in 81%, and retinal vasculitis in 56%. Cystoid macular edema was present in about a third of affected patients. Although uveitis is the most common cause of macular edema in MS patients, it must be recognized that fingolimod, an oral agent FDA approved for MS treatment, is itself associated with development in macular edema in approximately 0.05% of patients [22].

5970 patients with chronic anterior uveitis (CAA) from a different tertiary care uveitis referral center who were evaluated over a period of three decades were analyzed for underlying systemic disease [23]. Multiple sclerosis was present in 30 patients (1.6%). Notably, the percentage of patients with MS increased each decade and was associated with 2.36% of all new patients with CAA evaluated between 1995 and 2004. In a cross-sectional study of uveitis patients seen in Vienna between 1995 and 2009, the prevalence of MS was 0.9% [24]. Of the 25 cases of uveitis with MS described in that study, 19 were intermediate, 4 were anterior, and 2 were posterior. The mean age of these patients was 35.9 years but the gender distribution was not reported.

Uveitis may be associated with multiple types of neurological disease. A retrospective review of all patients seen in a tertiary care center with a diagnosis of uveitis identified 115 patients with an underlying neurological disease [25]. Fourteen of these patients, or 1% of the total number of patients seen in the referral center over the course of fifteen years, were diagnosed as having MS. Half of these patients (50%) carried a diagnosis of pars planitis and 29% had chronic granulomatous uveitis. Intermediate uveitis typically affects patients in the younger years, ranging from age 5 to 30, and does not typically have associations with either race or gender [26]. Bilateral disease is seen in at least 80%, and complications such as cataract, glaucoma, or cystoid macular edema are common [27].

## 4. What Is the Risk for Uveitis If MS Is Diagnosed?

Multiple groups have investigated the frequency of uveitis in patients with multiple sclerosis but the estimates vary widely from 0.4% to 28.5% depending on the study [28]. One study prospectively evaluated 50 MS patients, 61.8% of whom were female, with a complete eye examination in order to identify concomitant inflammatory eye disease [29]. None of the patients had a history of eye disease, including optic neuritis. Nine of the patients (18%), 4 female and 5 male, had retinal vascular changes, either venous sheathing or focal cuffing, often with fluorescein angiographic evidence of leakage and inflammation, without overt cells. Interestingly, a similar study was performed in Egypt in which 75 patients with MS were prospectively evaluated with a full ophthalmological examination [30]. This study differs from most studies on MS in that there was not a striking female preponderance (34 males, 41 females), although it is not clear if this reflected the gender distribution of patients in this country with MS. In this cohort, seven patients were diagnosed as having intermediate uveitis and, in contrast to other studies, five of the seven were male and all of the males were young, with a mean age of 20. A separate study performed in Croatia identified intermediate uveitis in 28.5% of a total of 42 patients with MS [31]. This is a very high prevalence of uveitis, higher than other studies, and it would be interesting to understand more about this patient population to determine why their risk for uveitis is so high.

A population based study of uveitis prevalence in 4300 patients in the Lyon Multiple Sclerosis cohort was performed using self-reporting of uveitis. The records of 31 identified patients were extracted and evaluated for uveitis classification, gender, and timing of MS onset. Three patients who either had Fuchs disease or HLA B27 associated disease were excluded from additional analysis. In the final cohort, 28 individuals were identified, 0.65% of the total number of MS patients. Women comprised 68% of the total patients with uveitis. In 46% of the patients, uveitis preceded the onset of MS. In this series 36% of patients had posterior uveitis whereas intermediate uveitis only was diagnosed in 7% of the patients [28]. Similarly, another review of 1098 patients from an MS clinic revealed a 1% prevalence of uveitis [32].

In a large population based study in the Northern California Kaiser Permanente Medical Care Program, a computer search of records of more than 5,000 patients with MS and age-matched controls was evaluated for prevalence of uveitis, among other autoimmune diseases [33]. Uveitis was diagnosed in 1.3% of patients with MS as compared to 0.6% of controls. Additional information about the demographics or type of uveitis is not available. In this series, uveitis preceded the diagnosis of MS in only 1% of patients. The population based studies on the prevalence of uveitis in MS are large and confirm that 0.65–1.3% of patients with MS will have uveitis. What is less clear is the timing of onset of uveitis relative to MS, with uveitis preceding MS diagnosis in anywhere from 1 to 46% of patients. These reports are somewhat limited by the quality of the available data but underscore the need to consider uveitis in patients with MS.

## 5. What Is the Risk of MS If Intermediate Uveitis (Pars Planitis) Is Diagnosed?

The term intermediate uveitis is a general one that describes uveitis in which the inflammatory cells are primarily located in the vitreous cavity. Pars planitis refers to a specific subset of idiopathic intermediate uveitis in which there is snowball or snowbank formation and no underlying systemic disease responsible for the inflammation [34]. Older studies did not exclude MS patients from the diagnosis of pars planitis, and in this section we therefore report series on intermediate uveitis and on pars planitis. The experience of a large tertiary care center with patients who carry a diagnosis of intermediate uveitis was published in 1993 [27]. In this retrospective study, 7% of patients with intermediate uveitis had MS. Another large series of 53 patients with pars planitis was reported in 1999 [35]. The mean age at diagnosis was 26 years with a range of 5 to 50 years. There were 20 males and 33 females. General medical or neurological data was available for 37 of the patients. Six were diagnosed with MS (11% of all intermediate uveitis patients), three prior to onset of uveitis. The patients with MS and pars planitis tended to be female (83%) and older (mean age of 36) and were also more likely to have vascular sheathing on examination. Another study of patients evaluated at a tertiary care uveitis center identified intermediate uveitis in 22.9% of patients (*n* = 438), and 10.3% of these patients had MS [20]. In another prospective clinical study of 21 patients with pars planitis, 47.6% demonstrated demyelinating lesions on MRI and 33.3% were diagnosed with definitive MS. Those patients diagnosed with MS were more likely to be older than the age of 25 [36]. This series reports the highest association with MS in patients with intermediate uveitis.

A population based study was performed in Olmsted, County, Minnesota, looking at all patients with a diagnosis of pars planitis who had been evaluated over a 20-year time period [37]. 25 patients with pars planitis who had sufficient medical records, provided authorization for the study, and did not have sarcoidosis or Behcet disease were identified. With longitudinal followup for a mean of 14.3 years, MS was diagnosed in 3 of the patients, for a rate of 12%.

Not surprisingly, there is geographic variation in rates of MS diagnosed in patients with intermediate uveitis. A recent study of 87 intermediate uveitis patients in a referral service in Tunisia revealed a lower percentage of patients with underlying MS (2.3%) and a higher association with sarcoidosis (9.2%) [38].

Genetic testing for risk factors in patients with intermediate uveitis reveals association with the IL2RA rs2104286 gene polymorphism [39]. Interestingly this same polymorphism is observed in MS and other autoimmune diseases.

Overall, in the majority of studies, 8–12% of patients with intermediate uveitis carry a diagnosis of or will develop MS. Additional risk factors for this diagnosis include female gender and bilateral disease. Although there is a gender disparity in rates of disease, these likely reflect the underlying increase in MS in females.

## 6. Possible Explanations for Gender Differences in MS

The gender differences in rates of MS are not understood, but there are many hypotheses with supporting data that suggest potential mechanisms [40]. There is a large body of literature that explores this topic, and only several of the leading hypotheses are presented here. One possibility is that these differences are mediated by epigenetic DNA modification by either hormonal or environmental stimuli [41]. Other studies implicate the X chromosome in disease susceptibility. For example, in the rodent MS model of experimental autoimmune encephalitis (EAE), having two X chromosomes increases disease susceptibility for unknown reasons [42]. Interestingly, in studies where the EAE disease was transferred from affected mice using purified T lymphocytes, T cells from female donors were more successful in creating disease in the recipient than were T cells from male donors, thus suggesting that there must be sexual differences in activation of the immune response [43]. There was also increased disease in female recipients in contrast to male recipients, implicating increased effector activation that is also determined by gender [44].

Hormonal differences between females and males may also impact disease severity and progression. Pregnancy is well documented to be associated with a decrease in MS relapses [7]. During pregnancy, the high expression of estriol, which is not measurable outside of pregnancy, may be associated with a milder course [26]. Phase II clinical trials are currently ongoing using exogenous estriol as a potential therapy in MS [45, 46]. Vitamin D has also been postulated to play a role in disease pathogenesis. Patients with MS typically have lower levels of vitamin D, and some studies report lower levels of vitamin D in females in comparison to males [47]. In EAE experiments, increasing vitamin D in the diet has been shown to ameliorate the disease [26].

## 7. Conclusion

These studies suggest that up to 3% of all patients evaluated in uveitis clinics also carry a diagnosis of MS. The uveitis observed in these patients is often bilateral. Intermediate uveitis is the most common, followed by anterior uveitis, which may be characterized by a granulomatous appearance. Retinal vascular changes in the absence of symptoms are seen in a significant percentage of patients. Patients who have known MS carry an approximately 1% risk of developing clinical intraocular inflammatory disease. Conversely, patients with intermediate uveitis are at moderate risk for MS, around 8–12%. The risk is higher in females and in those with bilateral disease. Therefore, these patients should be routinely questioned regarding transient (lasting several weeks) neurologic symptoms that might otherwise be ignored by the patient. Patients with uveitis and MS are more likely to be female, in concordance with the gender predisposition for MS. Importantly some cases of uveitis occur prior to the diagnosis of MS, and in these patients one must be particularly careful to be alert to the possible diagnosis, in particular if biologic immune modulators are contemplated for therapy, as exposure to TNF-alpha inhibitors heightens the risk for demyelinating lesions [48].
